# A Flexible Film Bulk Acoustic Resonator Based on β-Phase Polyvinylidene Fluoride Polymer

**DOI:** 10.3390/s20051346

**Published:** 2020-02-29

**Authors:** Ting Wu, Hao Jin, Shurong Dong, Weipeng Xuan, Hongsheng Xu, Leihe Lu, Zijing Fang, Shuyi Huang, Xiang Tao, Lin Shi, Shuting Liu, Jikui Luo

**Affiliations:** 1 Key Lab. of Advanced Micro/Nano Electronic Devices and Smart Systems of Zhejiang, College of Information Science and Electronic Engineering, Zhejiang University, Hangzhou 310027, China; 21731054@zju.edu.cn (T.W.); iseexuhs@zju.edu.cn (H.X.); lu_lei_he@163.com (L.L.); 21831080@zju.edu.cn (Z.F.); 11631009@zju.edu.cn (S.H.); 11531013@zju.edu.cn (X.T.); 11731035@zju.edu.cn (L.S.); 11531010@zju.edu.cn (S.L.); jackluo@zju.edu.cn (J.L.); 2 ZJU-Hangzhou Global Scientific and Technological Innovation Center, Hangzhou 311215, China; 3 Ministry of Education Key Laboratory of RF Circuits and Systems, Hangzhou Dianzi University, Hangzhou 310018, China; xuanweipeng@hdu.edu.cn

**Keywords:** bulk acoustic wave, β-phase PVDF polymer, flexible device, strain sensor, temperature sensor

## Abstract

This paper reports a novel flexible film bulk acoustic resonator (FBAR) based on β-phase polyvinylidene fluoride (PVDF) piezoelectric polymer. The proposed device was simulated and evaluated; then, a low-temperature photolithography process with a double exposure method was developed to pattern the electrodes for the device, which enabled the device to retain the piezoelectric properties of the β-phase PVDF film. Results showed that the β-phase PVDF FBARs had a resonant frequency round 9.212 MHz with a high electromechanical coupling coefficient ($k^{2}$) of 12.76% ± 0.56%. The device performed well over a wide bending-strain range up to 2400 με owing to its excellent flexibility. It showed good stability as a strain sensor with a sensitivity of 80 Hz/με, and no visible deterioration was observed after cyclic bending tests. The PVDF FBAR also exhibited an exceptionally large temperature coefficient of frequency (TCF) of −4630 ppm/K, two orders of magnitude larger than those of other FBARs based on common inorganic piezoelectric materials, extraordinarily high sensitivity for temperature sensing. All results showed that β-phase PVDF FBARs have the potential to expand the application scope for future flexible electronics.

## 1. Introduction

Flexible electronic technologies are very attractive for their use in many novel applications such as wearable electronics, implantable medical devices, and healthcare systems [1]. The flexible film bulk acoustic resonator (FBAR) is one such a technology with growing potential owing to its widespread applications in modern electronics and sensing. For example, FBAR devices are very promising for sensing and monitoring pressure [2], mass [3], ultraviolet (UV) light [4], and biomolecules [5] owing to their small size, high sensitivity, and low power consumption. Over the past several decades, a number of FBAR studies have focused on the development of novel materials (piezoelectric layer, substrate membrane) for the fabrication of high-performance FBARs [6], and flexible polymer substrates such as polyethylene terephthalate (PET) [7] and polyimide (PI) [8] have been successfully used in the fabrication of flexible FBAR devices. However, commonly used piezoelectric materials for FBAR devices (materials such as zinc oxide (ZnO), aluminum nitride (AlN), and lead zirconate titanate (PZT)) are inorganic, and are either brittle or not biocompatible. In addition, complex process combined with high-cost equipment for the deposit of these highly crystalline films can limit their widespread application and commercialization.

Polyvinylidine difluoride (PVDF), a well-known high-performance piezoelectric polymer, could be a strong candidate as a new flexible FBAR material due to its remarkable inherent flexibility, lightness, and robustness, as well as its high piezoelectric response and low acoustic impedance, similar to those of biological tissue. Since the discovery of the piezoelectric activity of the PVDF material by Kawai in 1969 [9], PVDF-based piezoelectric polymer has been used in a wide range of actuators and sensors, such as underwater acoustic transducers [10], ultrasonic inspection sensors [11], acceleration sensors [12], surface acoustic-wave devices [13], pressure sensors [14], and energy harvesters [15]. In addition, it has been utilized in medical and biological applications such as artificial muscles and organs [16], medical imaging [17], and blood-flow monitors [18]. However, a PVDF polymer cannot be processed by a standard lithography process, limiting its application in MEMS devices and electronics.

In this paper, we report on the feasibility of using a β-phase PVDF polymer for the development of FBARs. In this research, an FBAR device was designed with a 100 μm thick β-phase PVDF film sandwiched between two metal electrodes with a simulated resonant frequency of 9.418 MHz. A facile and reliable fabrication method was developed to successfully fabricate β-phase PVDF FBAR, which is compatible with the standard lithography process. The stability of the crystalline phase and the piezoelectricity of the β-phase PVDF film after the fabrication process were verified by X-ray diffraction (XRD) and piezoelectric coefficient $d_{33}$ measurement, respectively. The performance of the β-phase PVDF FBAR was analyzed and characterized both experimentally and theoretically. Furthermore, we conducted investigations on the performance of the β-phase PVDF FBAR for strain and temperature sensing, and results showed that the device has great potential for flexible applications in electronics and sensors. 

## 2. Modeling and Device Fabrication 

### 2.1. Numerical Modeling

**Figure 1:**
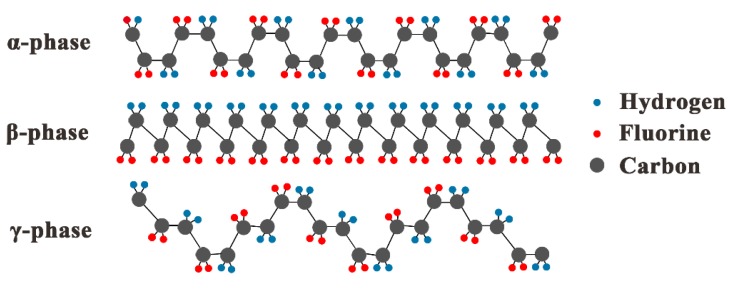
Structures of α, β, and γ phase polyvinylidene fluoride (PVDF).

Most PVDF properties, particularly piezoelectricity, are related to the strong electric dipole moment of the PVDF monomer unit (CH_2_-CH_2_-), owing to the electronegativity of fluorine atoms as compared to those of hydrogen and carbon atoms [19]. It is a kind of semicrystallized polymer with four crystalline structures (α, β, γ,and δ) that are defined by different chain conformations of the PVDF material [20]. The structures of most investigated and used crystalline PVDF phases are illustrated in Figure 1. Here, the β-phase with all trans (TTT) planar zigzag arrangement is essential for piezoelectric applications since it has the largest effective dipolar moment per unit cell, therefore exhibiting the highest piezoelectricity [21]. The PVDF used in this work is the polar β crystal phase.

A comparison of the relevant material parameters for common piezoelectric materials and β-phase PVDF is given in Table 1, showing that β-phase PVDF possesses outstanding mechanical flexibility and high piezoelectricity. As such, large displacement for PVDF material can be obtained due to its small Young’s modulus [22]. The very low acoustic impedance, as compared to that of inorganic piezoelectric materials, makes it useful as an acoustic sensor in water, organic materials, and biological tissue. Moreover, a comparatively high effective coupling coefficient is rather advantageous for wide bandwidth applications. It also shows a large temperature-dependence offset, long-term stability in dynamic condition, relatively simple fabrication process, and low cost for manufacturing in large volume [23]. 

**Figure 2:**
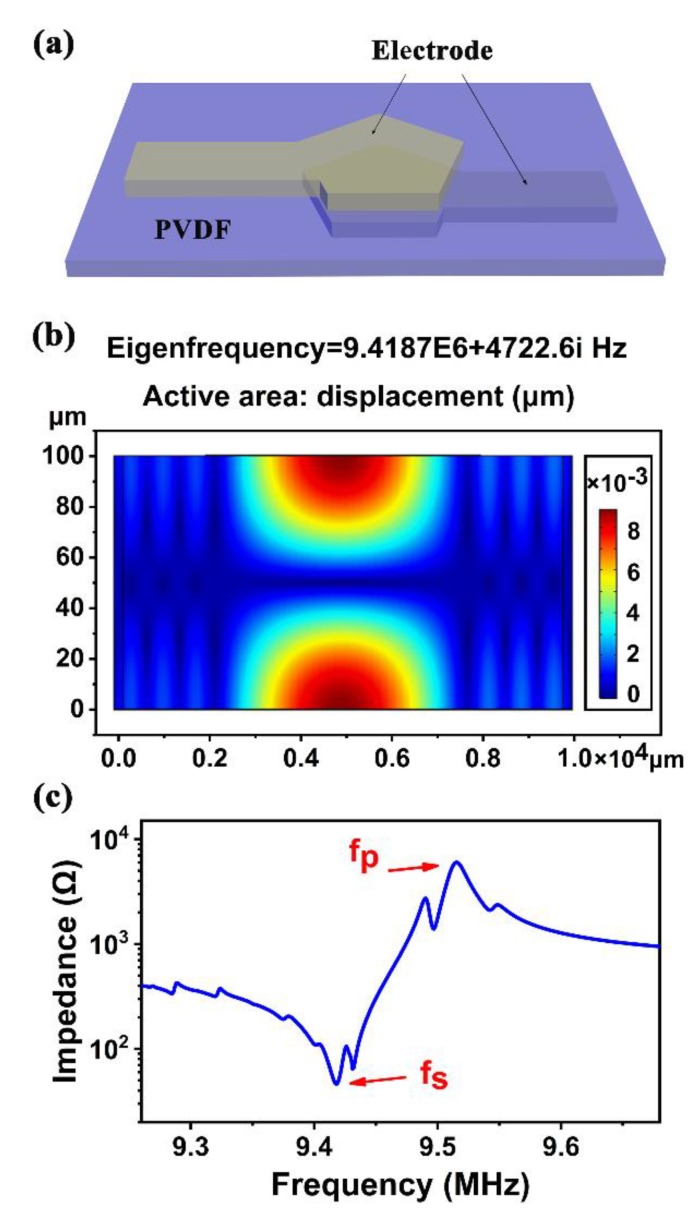
(**a**) Three-dimensional schematic of FBAR device structure; (**b**) simulated 2D cross-section displacement of β-phase PVDF FBAR in COMSOL; and (**c**) impedance of β -phase PVDF FBAR in COMSOL.

The β-phase PVDF FBAR was designed and analyzed by the finite-element method (FEM) using COMSOL Multiphysics before fabrication. For simplicity, we considered a device composed of a 100 μm thick PVDF piezoelectric layer sandwiched between top and bottom aluminum (Al) electrodes with 200 nm thickness, as shown in Figure 2a. For a stretched and poled piezoelectric PVDF film, its symmetry properties belonged to point group 2 mm [33], and in many cases, it is typically regarded as having isotropic elastic and dielectric constant; therefore, the elastic-compliance, piezoelectric-coefficient, and dielectric-permittivity matrices can be simplified as

(1) $$[\begin{array}{cc} \begin{array}{ccc} s_{11} & s_{12} & s_{13}\\ s_{21} & s_{22} & s_{23}\\ s_{31} & s_{32} & s_{33} \end{array} & \begin{array}{ccc} 0\; & 0\; & 0\\ 0\; & 0\; & 0\\ 0\; & 0\; & 0 \end{array}\\ \begin{array}{ccc} \;0\; & 0\; & 0\;\\ 0\; & 0\; & 0\;\\ 0\; & 0\; & 0\; \end{array} & \begin{array}{ccc} s_{44} & 0 & 0\;\\ 0 & s_{55} & 0\;\\ 0 & 0 & s_{66} \end{array} \end{array}]=\frac{1}{Y}[\begin{array}{cc} \begin{array}{ccc} 1 & -\sigma & -\sigma \\ -\sigma & 1 & -\sigma \\ -\sigma & -\sigma & 1 \end{array} & \begin{array}{ccc} 0\; & 0\; & 0\\ 0\; & 0\; & 0\\ 0\; & 0\; & 0 \end{array}\\ \begin{array}{ccc} 0\; & 0\; & 0\\ 0\; & 0\; & 0\\ 0\; & 0\; & 0 \end{array} & \begin{array}{ccc} 1+\sigma & 0 & 0\\ 0 & 1+\sigma & 0\\ 0 & 0 & 1+\sigma \end{array} \end{array}],\;\left[\begin{array}{cc} \begin{array}{ccc} 0 & 0 & 0\\ 0 & 0 & 0\\ d_{31} & d_{32} & d_{33} \end{array} & \begin{array}{ccc} 0 & d_{15} & 0\\ d_{24} & 0 & 0\\ 0 & 0 & 0 \end{array} \end{array}\right]\;\mathrm{and}\;\left[\begin{array}{ccc} \varepsilon_{11} & 0 & 0\\ 0 & \varepsilon_{22} & 0\\ 0 & 0 & \varepsilon_{33} \end{array}\right]=\varepsilon_{0}\varepsilon_{r}I_{3}$$

where Y is Young’s modulus, σ is Poisson’s ratio, $I_{3}$ is the identity matrix, $\varepsilon_{r}$ is the relative permittivity of PVDF, and $\varepsilon_{0}$ = 8.854 × 10^–12^ F/m is the permittivity of free space. The material parameters used in COMSOL are listed in Table 2. 

Under radio-frequency (RF) signal excitation, standing waves are generated in the PVDF film between the two electrodes. Figure 2b shows a typical resonant mode of the simulated FBAR device. The maximal displacement of particles in the device occurred at the bottom and top surfaces of the PVDF film, confirming that the β-phase PVDF film resonated in thickness mode. Figure 2c shows the simulated electrical impedance characteristic. Two well-defined resonant peaks can be seen at 9.418 and 9.515 MHz, where conductance and resistance show the local maxima, corresponding to the series and parallel resonance frequencies ($f_{s}$ and $f_{p}$), respectively. However, there were small peaks near the resonant frequencies, which could be attributed to the lateral acoustic waves, as shown in Figure 2b.

### 2.2. Device Fabrication

On the basis of the FEM analysis, PVDF FBARs were fabricated. The stable operating-temperature range for the β-phase PVDF polymer was −40 to 60 °C [36]. This is because high temperatures cause serious degradation of piezoelectric properties of the PVDF material due to dipole restoration to their original random positions in the PVDF film. For example, piezoelectric coefficient $d_{33}$ deteriorated by about 50% after being exposed to 100 ℃ for 1–4 h [36]. Thus, a key issue for fabricating working PVDF FBARs is to develop a relatively low-temperature fabrication process compared with those used to fabricate inorganic FBARs. Additionally, PVDF does not have chemical resistance to acetone, which is a traditional photoresist (PR) remover used in lift-off processes to produce metal electrodes. PVDF would therefore not be able to strip PR using acetone if the normal lift-off process were used to fabricate the PVDF-based FBARs. Although alternative fabrication methods, such as reactive ion etch-based PR removal [36], and screen-print and shadow-mask processes [37] were developed, these are either complex or not compatible with the standard lithography process for MEMS manufacturing. 

**Figure 3:**
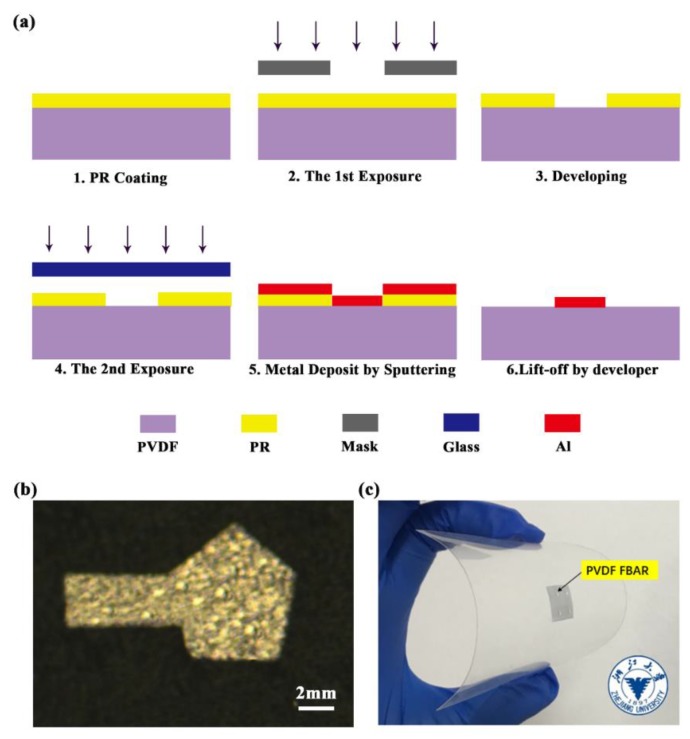
(**a**) Fabrication process for PVDF FBAR; (**b**) microscope image of fabricated electrode by double-exposure and lift-off process; (**c**) optical photo of fabricated FBAR device, showing its flexibility.

Here, we propose a novel double-exposure lift-off process to fabricate PVDF FBARs without using a high-temperature process and acetone solvent. The detailed process for patterning electrodes is illustrated in Figure 3a. Before fabrication, a 2 × 2 ${\mathrm{cm}}^{2}$ β-phase PVDF film (100 μm, Jinzhoukexin Electronic Materials Co., Ltd., Jinzhou, China) was prepared, and sequentially cleaned with isopropyl alcohol and deionized (DI) water. Next, AR-P5350 PR was coated on the top of the as-cleaned PVDF film with a thickness of ~2 μm. The prebake process was conducted at 60 ℃ for 1 h to cure the PR film. The wafer was then exposed to UV light with a dose of 25.6 $\mathrm{mJ}/{\mathrm{cm}}^{2}$ with a hard mask of electrode patterns, and developed in a developer (AR 300-26). The whole wafer was exposed to the same dose of UV light again with no mask, and then it was cleaned by $O_{2}$-plasma for 1 min to remove residual PR on the PVDF surface of the patterned areas. Then, aluminum was sputtered on top of the PVDF with a thickness of 200 nm. Both the top and bottom Al electrodes were deposited by RF magnetron sputtering and patterned by the double-exposure UV-photolithography process. Instead of acetone, the developer was used to remove the remaining PR and unwanted aluminum to form the electrodes. The patterned electrodes can be seen by the optical microscope, as shown in Figure 3b. The back-side electrode was fabricated in the same way. Figure 3c shows a photograph of the fabricated FBAR device on a PET film, which exhibits excellent flexibility. 

Once fabricated, the electrode pads of the FBAR were connected to a printed circuit board (PCB) via wires using silver paste with 50 Ω match impedance. Outputs from the PCB were connected to a vector network analyzer (Agilent E5071C) for characterizing its transmission properties. A LabVIEW-based program was developed to implement automated measurements of the frequency shift of the device.

## 3. Results and Discussion

### 3.1. Device Characterization

**Figure 4:**
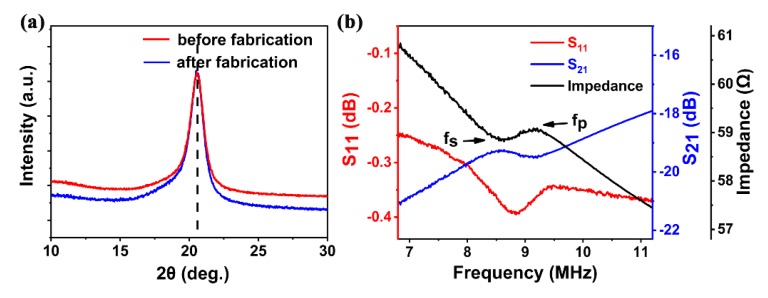
(**a**) Comparison of X-ray diffraction patterns of β-phase PVDF films before and after fabrication, showing no visible influence from fabrication processes; (**b**) S-parameter and impedance characteristic curve of FBAR device.

The crystallinity of the PVDF film was characterized using XRD as shown in Figure 4a. A well-defined peak at 2θ = 20.34° is characteristic of the sum of the diffraction at the (200) and (110) crystalline planes of the β-phase PVDF film. The position of the peaks of the β-phase PVDF was observed to be consistent after the fabrication process, clearly showing that there was no noticeable deterioration and damage caused by the process to the piezoelectric properties. This demonstrates the stability and consistency of the fabrication process.

Figure 4b shows the reflection ($S_{11}$) and transmission ($S_{21}$) characteristics of the fabricated β-PVDF FBAR, as well as the impedance curve obtained from the measured S-parameter. Although the resonant peak of the FBAR is relatively weak and broad, it clearly showed resonance with $f_{s}$ of about 8.708 MHz and $f_{p}$ of about 9.212 MHz. The difference between simulated ($f_{s}$ of 9.418 MHz, $f_{p}$ of 9.515 MHz) and measured results was probably due to the use of ideal material constants of β-phase PVDF in COMSOL based simulation, while the practical material is nonideal, with a rough surface (Ra ~170 nm) and a high degree of porosity inside the film [38] that cause high mechanical losses (low mechanical Q) [32] and degradation of the electrical properties of the material (lower dielectric constant) [39]. The nonideal material properties and thick film could also be responsible for the weak resonance observed. The electromechanical coupling coefficient ($k^{2}$) was directly extracted from these measurements via Equation (2) to be 12.76% ± 0.56% with the scattering of *f*_s_ and *f*_p_ being considered, much higher than those of FBARs made of inorganic piezoelectric materials. The high electromechanical coupling of the β-phase PVDF is owing to its high piezoelectric coefficient $d_{33}$.

(2) $$k^{2}=\frac{\pi^{2}}{4}\left(\frac{f_{s}}{f_{p}}\right)\frac{f_{p}-f_{s}}{f_{p}}$$

To further clarify whether β-phase PVDF properties deteriorated as a result of the fabrication process, we measured piezoelectric coefficient $d_{33}$ using a ZJ-3A quasistatic $d_{33}$ measurement machine (LACAS, Beijing, China). The values of $d_{33}$ before and after the fabrication process were 27 and 26 pC/N, respectively, well within the experimental scatter, indicating little influence from the fabrication process. Such strong piezoactivity after the fabrication process makes it practically useful in many applications. 

### 3.2. Strain Response

**Figure 5:**
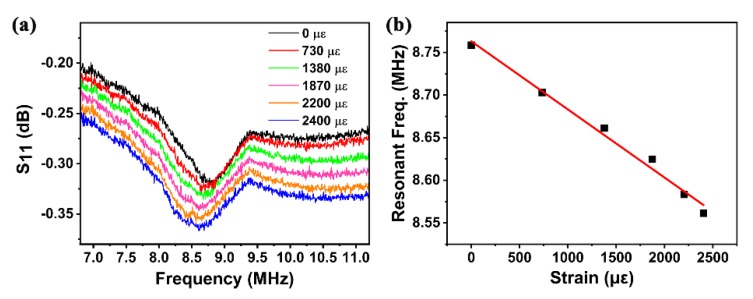
(**a**) Reflection spectra of device under different bending strains; (**b**) frequency shift as device strain function.

The performance of the FBAR device as a strain sensor was investigated with a bending test. A robotic arm (HSV-500) was used to bend the device, and a network analyzer was utilized to measure the strain response. Figure 5a shows the reflection spectra of the device under different strains. The device performed well over a wide strain range of up to 2400 με (μ is the unit prefix of $10^{-6},\;\varepsilon$ represents the applied strain), demonstrating the excellent flexibility of the β-phase PVDF FBAR device. Good negative linearity of the resonant frequency ($f_{s}$) vs. strain was obtained, as shown in Figure 5b. Sensitivity (defined as $S_{sen}=\Delta f/\Delta s$) was calculated to be 80 Hz/με, with a linearity of 0.988, excellent for strain sensing.

The reasons for the negative linearity of resonant frequency ($f_{s}$) vs. strain are not clear. Elastic moduli changing under strain could be a factor that induces the frequency shift of FBARs due to the deterioration of the elastic properties of the material under strain [40]. Another possible reason for the negative linearity is the changes of atom structure and crystalline phases of the PVDF polymer under stress. It is well known that the piezoelectric properties of PVDF polymer are strongly dependent on its β crystalline phase, and these properties are responsible for piezoelectric activity and higher longitudinal acoustic velocity. However, β-phase content and degree of crystallinity are dependent on the zigzag trans (TTT) planar arrangement, as shown in Figure 1, and thus are strongly influenced by stretch ratio [35]. When strain is applied, the crystalline structure and crystal phase may change; these changes extend the acoustic propagation path and reduce velocity, and frequency decreases with the increase of strain. However, more work is necessary to clarify this behavior. 

**Figure 6:**
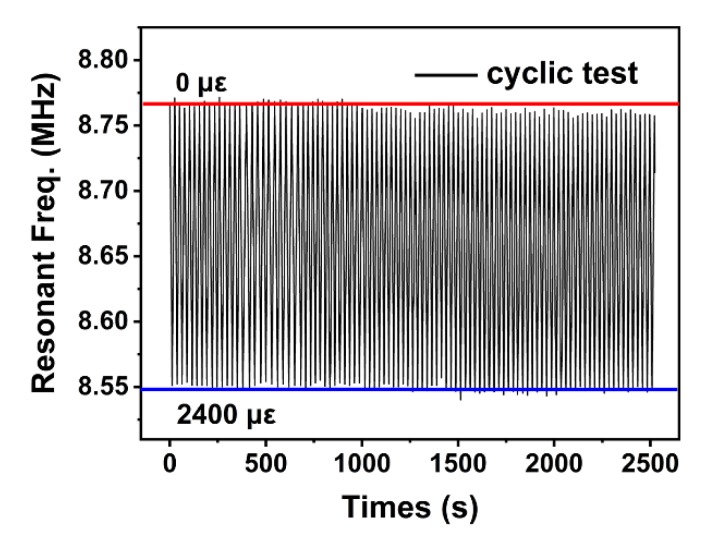
Frequency shift under cyclic bending strain from zero to 2400 με for 98 cycles (2500 s), showing excellent device stability.

Figure 6 shows the cyclic bending test result of up to 98 times (2500 s), with bending strain varying cyclically from zero to 2400 με. The average resonant-frequency shift was from 8.769 to 8.549 MHz when bending strain changed from zero to 2400 με. The resonant frequency of the overall bending test drifted slightly downward due to the slight position shift of the robotic arm and the decrease of wire bonding strength. Nevertheless, results showed that the FBAR device has excellent stability and favorable fatigue properties, indicating its good potential for flexible strain sensors. 

### 3.3. Temperature Sensing 

It is well known that many ambient quantities may affect FBAR performance, with temperature being one of the main concerns. In order to improve the performance of FBAR devices, a large part of research on FBAR temperature characteristics has focused on how to reduce the effect of temperature fluctuation, such as by adding a silicon dioxide compensation layer [41], embedding a microheater layer between the supporting layer and piezoelectric layer, or stiffening (mechanically or electrically) the piezoelectric film [42]. However, an alternative approach could be utilizing the effect of temperature on FBAR to fabricate FBAR temperature sensors, for which there is a huge market [43]. 

**Figure 7:**
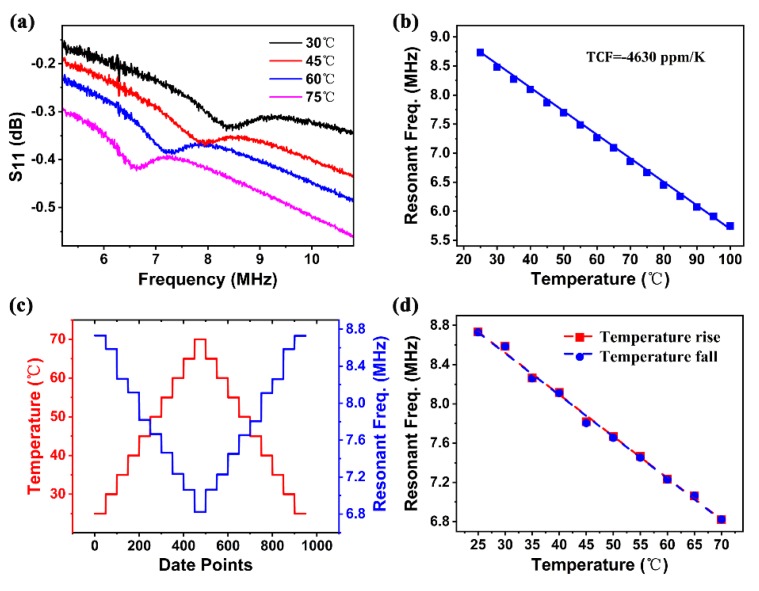
(**a**) Reflection spectra of device under different temperatures; (**b**) resonant frequency as function of temperature, showing temperature coefficient of −4630 ppm/K frequency, calculated from gradient; (**c**) frequency at each temperature test point for 50 times; (**d**) frequency shift under cyclic temperature scanning, showing good consistency with no visible hysteresis.

Figure 7a shows the frequency response of the PVDF FBAR to temperature variation. When temperature increased, the reflection spectrum accordingly shifted to the left. The temperature coefficient of frequency (TCF) is normally used to assess the temperature dependence of the device resonant frequency, which is defined by Equation (3).

(3) $$\mathrm{TCF}=\frac{1}{f_{0}}\frac{\Delta f}{\Delta T}\times 10^{6}\;\left[\mathrm{ppm}/K\right],$$

where $f_{0}$, Δf, and ΔT are the original resonant frequency, shift of the resonant frequency, and change of temperature, respectively. Figure 7b shows the resonant-frequency ($f_{s}$) shift of the FBAR device as a function of temperature. Excellent linearity of 0.999 was achieved in the measurement temperature range from 25 to 100 °C. From this result, Δf/ΔT = 0.0405 MHz/K was obtained, and the measured resonant frequency of 8.75 MHz of the FBAR at $T_{0}$ = 25 °C was used as $f_{0}$. The TCF of the device was therefore calculated to be −4630 ppm/K, which is about two orders of magnitude larger than those of common inorganic-piezoelectric-material-based FBARs (about −25 to 60 ppm/K) [44] owing to the high thermal expansion coefficient of the β-phase PVDF material, as shown in Table 1. Hence it can be exploited efficiently in the area of high sensitivity temperature sensors.

We also carried out a cyclic temperature test to clarify temperature stability and repeatability. The device was suspended and end-anchored in a hot-air oven, which ensured that the device was heated evenly and avoided errors possibly induced by strain-related frequency shift. The LabVIEW-based program used to implement automated measurements could extract the resonant frequency by smoothing the curve, and find the peak by comparing the surrounding points. For accuracy measurement, resonant frequency at each temperature test point was obtained from sweeping repeated 50 times. Figure 7c shows that frequency was stable and repeatable at each temperature test point, and frequency values were almost the same (with a small difference of ±0.005 MHz) when temperature increased and then returned to the original point for the temperature range of 25–70 ℃, as shown in Figure 7d, demonstrating the device’s excellent stability and reliability, and its great potential for the application of flexible temperature sensors.

Although β-phase PVDF FBAR had a weak piezoelectric response, it performed well as a flexible sensor. The existence and optimization of these properties are intimately related to the fraction of the polymer in the crystalline phase, particularly its structure, microstructure, and orientation. All of these, in turn, heavily depend on processing conditions. Nowadays, much research is carried out with the aim of improving the quality of β-phase PVDF films. Examples of proposed solutions so far include different polarization methods to obtain well-defined β-phase crystals [45], and suitable quenching temperature and PVDF concentration to reduce porosity [46]. Our research aims to further this work, and we anticipate better β-phase PVDF films in the future to achieve better performance and extend its application area.

## 4. Conclusions

In conclusion, a flexible FBAR was developed by using a β-phase PVDF polymer as the piezoelectric material. The performance of this device was analyzed and characterized both experimentally and theoretically. A facile low-temperature lithography process with double exposure was developed to successfully pattern the electrodes on the β-phase PVDF film without deteriorating the properties of the β-phase PVDF polymer and the FBAR devices. The series and parallel resonance frequencies ($f_{s}$ and $f_{p}$) experimentally measured were 8.708 and 9.212 MHz, respectively, and the high obtained $k^{2}$ of 12.76% ± 0.56% is rather advantageous for wide bandwidth applications. For the bending test, results showed that the device could handle a wide strain range of up to 2400 με, and that it was capable of strain sensing with sensitivity and linearity of 80 Hz/με and 0.988, respectively. The cyclic bending test showed the device’s excellent mechanical stability and reliability. An exceptionally large TCF of −4630 ppm/K was obtained, exhibiting the device’s excellent potential for high-sensitivity temperature-sensing applications. All results demonstrated that the β-phase PVDF FBAR merits further study and development, and is promising in the domain of future flexible electronics. 

## Figures and Tables

**Table 1:** Comparison of piezoelectric materials.

Materials	ZnO [24,25,26,27,28]	AlN [24,25,26,28,29]	PZT [26,28,30]	β-PVDF [28,31,32,33,34,35]
$\mathrm{Density}\;(g/cm^{3}$)	5.61	3.26	7.55	1.78
Acoustic velocity (m/s)	6340	11,350	3603	2200
Young′s Modulus (GPa)	210	320	1200	3
$\mathrm{Acoustic}\;\mathrm{Impedance}\;(10^{6}\;kg/m^{2}s)$	36.2	37	27.2	2.7
$\mathrm{Effective}\;\mathrm{coupling}\;\mathrm{coefficient}.k_{eff}^{2}\;$(%)	7.8	6.0	20.25	14
$\mathrm{Piezoelectric}\;\mathrm{coefficient}\;d_{33}\;\left(pC/N\right)$	12	4.5	289–380	−24 to 34
$\mathrm{Coefficient}\;\mathrm{of}\;\mathrm{thermal}\;\mathrm{Expansion}\;(CTE,\;10^{-6}$)	4	5.2	1.75	42–75

**Table 2:** Constants used to define β-phase PVDF flexible film bulk acoustic resonator (FBAR) in COMSOL.

Parameter	Value
**Y**	$3\;\times \;10^{9}\;N/m^{2}$
**σ**	0.4
**ρ**	$1.78\;g/{\mathrm{cm}}^{3}$
**$d_{31}$**	$18\;\times 10^{-12}\;C/N$
**$d_{32}$**	$5\;\times 10^{-12}$ C/N
**$d_{33}$**	$-26\;\times 10^{-12}$ C/N
**$\varepsilon /\varepsilon_{0}$**	9.5
**$tan\delta_{e}$**	0.02

## References

[1] William W., Salleo A. (2009). Flexible Electronics: Materials and Applications.

[2] Gu C., Xuan W., Dong S., Wang X., Li H., Yu L., Luo J. (2018). Temperature calibrated on-chip dual-mode film bulk acoustic resonator pressure sensor with a sealed back-trench cavity. J. Micromech. Microeng..

[3] Johnston M.L., Kymissis I., Shepard K.L. (2010). FBAR-CMOS oscillator array for mass-sensing applications. IEEE Sens. J..

[4] Qiu X., Tang R., Zhu J., Oiler J., Yu C., Wang Z., Yu H. (2011). The effects of temperature, relative humidity and reducing gases on the ultraviolet response of ZnO based film bulk acoustic-wave resonator. Sens. Actuators B Chem..

[5] Nirschl M., Rantala A., Tukkiniemi K., Auer S., Hellgren A.-C., Pitzer D., Schreiter M., Vikholm-Lundin I. (2010). CMOS-Integrated Film Bulk Acoustic Resonators for Label-Free Biosensing. Sensors.

[6] Chen J., Guo H., He X., Wang W., Xuan W., Jin H., Dong S., Wang X., Xu Y., Lin S. (2015). Development of flexible ZnO thin film surface acoustic wave strain sensors on ultrathin glass substrates. J. Micromech. Microeng..

[7] Hu N.N., He X.L., Bian X.L., Chen G.H., Dong S.R., Luo J.K. Novel flexible FBAR on PET substrate. Proceedings of the 2014 IEEE International Conference on Electron Devices and Solid-State Circuits.

[8] Chen G., Zhao X., Wang X., Jin H., Li S., Dong S., Flewitt A.J., Milne W.I., Luo J.K. (2015). Film bulk acoustic resonators integrated on arbitrary substrates using a polymer support layer. Sci. Rep..

[9] Kawai H. (1969). The Piezoelectricity of Poly (vinylidene Fluoride). Jpn. J. Appl. Phys..

[10] Uma G., Umapathy M., Jose S., Natarajan V., Kathiresan M. (2007). Design and Simulation of PVDF-MOSFET Based MEMS Hydrophone. Instrum. Sci. Technol..

[11] Brown L.F., Carlson D.L. (1989). Ultrasound transducer models for piezoelectric polymer films. IEEE Trans. Ultrason. Ferroelectr. Freq. Control..

[12] Schulze R., Gessner T., Heinrich M., Schueller M., Forke R., Billep D., Sborikas M., Wegener M. Integration of piezoelectric polymer transducers into microsystems for sensing applications. Proceedings of the Proceedings of ISAF-ECAPD-PFM 2012.

[13] Kumar A., Thachil G., Dutta S. (2019). Ultra high frequency acoustic wave propagation in fully polymer based surface acoustic wave device. Sens. Actuators A Phys..

[14] Shirinov A.V., Schomburg W.K. (2008). Pressure sensor from a PVDF film. Sens. Actuators A Phys..

[15] Sun C., Shi J., Bayerl D.J., Wang X. (2011). PVDF microbelts for harvesting energy from respiration. Energy Environ. Sci..

[16] Preethichandra D.M., Kaneto K. Bending curvature measurement using a SAW sensor fabricated on a polyvinylidine difluoride (PVDF) substrate. Proceedings of the International Conference on Sensing Technology.

[17] Gallantree H.R. (1983). Review of transducer applications of polyvinylidene fluoride. IEE Proc. I-Solid-State Electron Devices.

[18] Chang W.-Y., Chu C.-H., Lin Y.-C. (2008). A flexible piezoelectric sensor for microfluidic applications using polyvinylidene fluoride. IEEE Sens. J..

[19] Nalwa H.S. (1995). Ferroelectric Polymers: Chemistry: Physics and Applications.

[20] Martins P., Lopes A.C., Lanceros-Mendez S. (2014). Electroactive phases of poly (vinylidene fluoride): Determination, processing and applications. Prog. Polym. Sci..

[21] Correia H.M., Ramos M.M. (2005). Quantum modelling of poly (vinylidene fluoride). Comput. Mater. Sci..

[22] Kaneko R., Froemel J., Tanaka S. (2018). Development of PVDF-TrFE/SiO2 composite film bulk acoustic resonator. Sens. Actuators A Phys..

[23] Je S.-S., Sharma T., Lee Y., Gill B., Zhang J.X. A thin-film piezoelectric PVDF-TrFE based implantable pressure sensor using lithographic patterning. Proceedings of the 2011 IEEE 24th International Conference on Micro Electro Mechanical Systems.

[24] Bertram A.A. (1973). Acoutic Fields and Wves in Solid.

[25] Chi-Jung C. (2012). Design, Modeling and Fabrication of Shear Mode Bulk Acoustic Wave Sensor as a Potential Biosensor.

[26] Hao J. (2006). The Study of Thin Fim Bulk Acoustic Wave Resonators (FBAR) Technology.

[27] Qing-Xin S., Kirby P., Eiju K., Masaaki I., Qi Z., Roger W. (2001). Thin-Film Bulk Acoustic Resonators and Filters Using ZnO and Lead–Zirconium–Titanate Thin Films. IEEE Trans. Microwave Theory Tech..

[28] Fu Y.Q., Luo J.K., Nguyen N.T., Walton A.J., Flewitt A.J., Zu X.T., Li Y., McHale G., Matthews A., borra E.I. (2017). Advances in piezoelectric thin films for acoustic biosensors, acoustofluidics and lab-on-chip applications. Prog. Mater. Sci..

[29] Verdu Tirado J. (2010). Bulk Acoustic Wave Resonators and their Application to Microwave Devices.

[30] Huidong L., Daniel D., Thomas J.C. (2012). Piezoelectric Materials Used in Underwater Acoustic Transducers. Sens. Lett..

[31] Measurement Specialties, Inc. (1999). Piezo Film Sensors Technical Manual.

[32] Eovino B.T. (2015). Design and Analysis of a PVDF Acoustic Transducer towards an Imager for Mobile Underwater Sensor Networks.

[33] Zhang Q.M., Bharti V., Kavarnos G., Schwartz M. (2002). Poly (Vinylidene Fluoride) (PVDF) and its Copolymers. Encyclopedia of Smart Materials.

[34] Ueberschlag P. (2001). PVDF piezoelectric polymer. Sens. Rev..

[35] Gomes J., Serrado Nunes J., Sencadas V., Lanceros-Mendez S. (2010). Influence of the β-phase content and degree of crystallinity on the piezo- and ferroelectric properties of poly(vinylidene fluoride). Smart Mater. Struct..

[36] Han H., Nakagawa Y., Takai Y., Kikuchi K., Tsuchitani S., Kosimoto Y. (2012). Microstructure fabrication on a β-phase PVDF film by wet and dry etching technology. J. Micromech. Microeng..

[37] Kärki S., Lekkala J. (2009). A new method to measure heart rate with EMFi and PVDF materials. J. Med Eng. Technol..

[38] Sencadas V., Gregorio Filho R., Lanceros-Mendez S. (2006). Processing and characterization of a novel nonporous poly(vinilidene fluoride) films in the β phase. J. Non-Cryst. Solids.

[39] Eberle G., Schmidt H., Eisenmenger W. (1996). Piezoelectric polymer electrets. IEEE Trans. Dielectr. Electr. Insul..

[40] Jan W., Mathias L., Robert P., Dana P., Matthias S. Sensor for Ambient Pressure and Material Strains using a Thin Film Bulk Acoustic Resonator. Proceedings of the IEEE Ultrasonics Symposium.

[41] Yu H., Pang W., Zhang H., Kim E.S. (2007). Ultra temperature-stable bulk-acoustic-wave resonators with SiO 2 compensation layer. IEEE Trans. Ultrason. Ferroelectr. Freq. Control..

[42] Bin Z., Yang G., Yi H.E. (2014). Analysis of the FBAR temperature-frequency drift characteristics. Piezoelectics Acoustooptics.

[43] García-Gancedo L., Pedros J., Zhao X.B., Ashley G.M., Flewitt A.J., Milne W.I., Ford C.J.B., Lu J.R., Luo J.K. (2012). Dual-mode thin film bulk acoustic wave resonators for parallel sensing of temperature and mass loading. Biosens. Bioelectron..

[44] Pensala T. (2011). Thin Film Bulk Acoustic Wave Devices: Performance Optimization and Modeling.

[45] Nunes J.S., Sencadas V., Wu A., Kholkin A.L., Vilarinho P.M., Lancerosméndez S. (2006). Electrical and Microstructural Changes of β-PVDF under Different Processing Conditions by Scanning Force Microscopy. Mrs Proc..

[46] Soin N., Boyer D., Prashanthi K., Sharma S., Narasimulu A.A., Luo J., Shah T.H., Siores E., Thundat T. (2015). Exclusive self-aligned β-phase PVDF films with abnormal piezoelectric coefficient prepared via phase inversion. Chem. Commun..

